# Esmolol does not improve quality of postsurgical recovery after ambulatory hysteroscopy: A prospective, randomized, double-blinded, placebo-controlled, clinical trial

**DOI:** 10.1097/MD.0000000000012647

**Published:** 2018-10-12

**Authors:** Gildasio S. De Oliveira, Mark C. Kendall, Robert J. McCarthy

**Affiliations:** a Department of Anesthesiology, Rhode Island Hospital, Alpert School of Medicine, Brown University, Providence, RI; b Department of Anesthesiology, Rush School of Medicine, Chicago, IL; c Department of Healthcare Policy and Practice, School of Public Health, Brown University, Providence, RI.

**Keywords:** esmolol, pain, recovery, surgery

## Abstract

**Introduction:**

Intraoperative systemic esmolol has been shown to reduce postsurgical pain. Nonetheless, it is unknown whether the use of intraoperative systemic esmolol can improve patient-reported postsurgical quality of recovery. The main objective of the current investigation was to evaluate the effect of intraoperative esmolol on postsurgical quality of recovery. We hypothesized that patients receiving intraoperative esmolol would report better quality of postsurgical recovery than the ones receiving saline.

**Methods:**

The study was a prospective randomized double-blinded, placebo-controlled, clinical trial. Healthy female subjects undergoing outpatient hysteroscopic surgery under general anesthesia were randomized to receive intravenous esmolol administered at a rate of 0.5 mg/kg bolus followed by an infusion of 5 to 15 μg/kg/min or the same volume of saline. The primary outcome was the Quality of Recovery 40 (QOR-40) questionnaire at 24 hours after surgery. Other data collected included postoperative opioid consumption and pain scores. Data were analyzed using group *t* tests and the Wilcoxon exact test.

**Results:**

Seventy subjects were randomized and 58 completed the study. There was not a clinically significant difference in the global QoR-40 scores between the esmolol and saline groups at 24 hours, median (interquartile range) of 179 (171–190) and 182 (173–189), respectively, *P* = .82. In addition, immediate post-surgical data in the post-anesthesia care unit did not show a benefit of using esmolol compared to saline in regard to pain scores, morphine consumption, and postoperative nausea and vomiting.

**Conclusions:**

Despite current evidence in the literature that intraoperative esmolol improves postsurgical pain, we did not detect a beneficial effect of intraoperative esmolol on patient-reported quality of recovery after ambulatory surgery. Our results confirm the concept that the use of patient-centered outcomes rather than commonly used outcomes (e.g., pain scores and opioid consumption) can change the practice of perioperative medicine.

## 1 Introduction

Women having outpatient surgery have worse quality of postoperative recovery when compared to male couteparts.^[1,2]^ It has demonstrated that some analgesic intraoperative interventions can improve patient-reported post-surgical quality of recovery, whereas others do not alter quality of recovery.^[3–6]^ Nonetheless, many intraoperative interventions that are known to improve post-surgical analgesia have not yet been tested to detect whether they also have an effect on patient-reported quality of post-surgical recovery.^[7–9]^ This is important as a focus on patient-centered care has been shown to improve outcomes and reduce costs.^[10,11]^

Many studies have consistently demonstrated a post-surgical analgesic benefit of using intraoperative esmolol.^[12,13]^ Hippocampal activation during stressful situations can augment nociception through the stimulation of n-methyl-d-aspartate receptors.^[14,15]^ It is therefore hypothesized that the blockage of beta-adrenergic receptors in the hippocampus by esmolol may attenuate perceived pain intensity. In contrast to the post-surgical analgesic effects of intraoperative esmolol, no study has examined the effect of intraoperative esmolol on patient reported post-surgical quality of recovery. This knowledge would help to determine whether the analgesic effect of intraoperative esmolol is clinically significant for post-surgical patients.

The main objective of the current investigation is to evaluate the effect of esmolol on patient reported quality of recovery after ambulatory gynecological surgery. We hypothesized that subjects receiving intraoperative esmolol would report better quality of post-surgical recovery when compared to the ones receiving saline.

## 2 Methods

This study was a prospective, randomized, double-blinded placebo-controlled trial. Clinical trial registration for this study can be found at ClinicalTrial.gov; url: http: //www.clinicaltrials.gov; registration identified:NCT01782898. Study approval was obtained from the Northwestern University Institutional Review Board, and written informed consent was obtained from all the study participants.

Eligible subjects were healthy females undergoing outpatient gynecologic hysteroscopic surgery. Patients with a history of chronic use of opioids, liver disease, and/or pregnant subjects were not enrolled. Reasons for exclusion from the study following study drug administration were surgeon or patient request. Subjects were randomized using a computer-generated table of random numbers into 2 groups to receive intravenousesmolol administered at a rate of 0.5 mg/kg bolus followed by an infusion of 5 to 15 μg/kg/min or the same volume of saline. The dose of esmolol is consistent with previous studies that demonstrated the efficacy of the drug to ameliorate post-surgical pain.^[16,17]^ The drug was prepared and dispensed by the hospital pharmacy and was identical for both study groups. A research associate blinded to the group allocation was responsible to collect all the data for the study.

All subjects were premedicated with 0.02 to 0.04 mg/kg IV midazolam and propofol 1 to 2 mg/kg was administered for anesthesia induction. A laryngeal mask airway was inserted by an anesthesia resident physician or a certified registered nurse anesthetist under supervision of an attending anesthesiologist. Anesthesia maintenance was achieved using fentanyl (25 μg every 5 minutes to keep blood pressure within 20% of baseline) and sevoflurane titrated to a Bispectral index (Aspect Medical System Inc, Norwood, MA) between 40 and 60. At the end of the surgical procedure, all subjects received intravenous ondansetron 4 mg and 30 mg of intravenous ketorolac.^[18,19]^

In the post-anesthesia care unit (PACU), subjects were asked to rate their pain at rest upon arrival and at regular intervals on a 0 to 10 pain numeric rating scale (NRS), where 0 means no pain and 10 is the worst pain imaginable. The area under the NRS pain scale versus time curve was calculated using the trapezoidal method as an indicator of pain burden during early recovery (Graph Pad Prism ver 5.03, Graph Pad Software, Inc., La Jola, CA). Intravenous hydromorphone was administered every 5 minutes to maintain an NRS pain score of <4 of 10. In cases of postoperative nausea or vomiting, subjects received 10 mg IV metoclopramide, followed by 5 mg IV prochlorperazine if necessary. At discharge, subjects were instructed to take a combination of hydrocodone 10 mg plus acetaminophen 325 mg q 6 hours for pain >4 of 10. Postoperative opioid consumption was converted to an equivalent dose of oral morphine.^[20]^

Subjects were contacted by telephone 24 hours after the procedure by a research associate unaware of group allocation and were questioned regarding thier analgesic consumption, pain score, and the QoR-40 questionnaire was administered.^[21]^ The questionnaire consists of 40 questions that examine 5 domains of patient recovery using a 5-point Likert scale: none of the time, some of the time, usually, most of the time, and all of the time. The 5 domains include physical comfort, pain, physical independence, psychological support, and emotional state. Individualized items of the questionnaire have been previously presented by our group.^[22]^ Other perioperative data collected included subject's age, height, weight, American Society of Anesthesiologist physical status, surgical duration, intraoperative remifentanil use, total intravenous fluids, total amount of hydromorphone in PACU, and total oral opioid consumption at home.

The primary outcome was the global QoR-40 score. Global QoR-40 scores range from 40 to 200 for representing, respectively, very poor to outstanding quality of recovery. A sample size of 31 subjects per group was estimated to achieve 80% power to detect a 10-point difference in the aggregated QoR-40 score for the 2 study groups to be compared assuming an overall standard deviation of 14 similar what was observed in a previous investigation.^[23,24]^ A 10-point difference represents a clinically relevant improvement in quality of recovery based on previously reported values on the mean and range of the QoR-40 score in patients following anesthesia and surgery.^[25]^ To compensate for drop-outs and losses to follow-up, 70 subjects were recruited and randomized. The sample size calculation was made using PASS version 13 (NCSS, LLC, Kaysville, UT).

The Shapiro-Wilk and Anderson-Darling tests were used to test the assumption of normal distribution (*P* > .1). Normally distributed interval data are reported as mean (SD) and were evaluated with 2-group *t* test with unequal variance assumed. Non-normally distributed interval and ordinal data are reported as median (range or interquartile range [IQR]) and compared among groups using the Wilcoxon exact test. Categorical data were compared using Fisher exact test. The criterion for rejection of the null hypothesis comparisons for the primary outcome was *P* < .05and *P* < .01 for other secondary outcomes. All reported *P* values are 2-tailed. Statistical analysis was performed using Stata version 13 (College Station, TX).

## 3 Results

The details of the conduct of the study are shown in the consort flow diagram. Seventy subjects were randomized and 58 completed the study. Patients were enrolled consecutively from March 2014 through March 2015. Patients’ baseline characteristics and surgical factors were not different between study groups (Table 1).

**Table 1:**
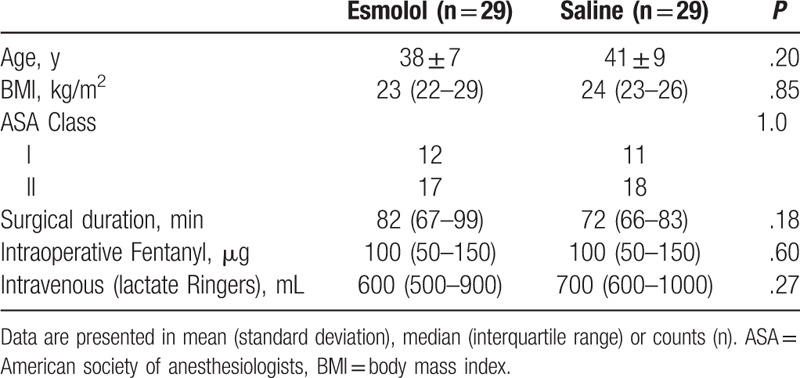
Baseline demographics and surgical characteristics.

Immediate post-surgical (PACU) data did not show a benefit of using esmolol compared to saline in regard to pain scores, morphine consumption, and postoperative nausea and vomiting (Table 2). Similarly, there was not a clinically significant difference in the global QoR-40 scores between the esmolol and the saline groups at 24 hours, median (IQR) of 179 (171–190) and 182 (173–189), respectively, *P* = .82. There was also no clinical difference between the study groups in any of the subcomponent items of the QoR-40 (Table 3). Subjects in the esmolol group required a median (IQR) of 0 (0 to 10) (oral mg of morphine equivalents) compared to 0 (0–20) (oral mg of morphine equivalents) in the saline group, (*P* = .33) over 24 hours after hospital discharge.

**Table 2:**
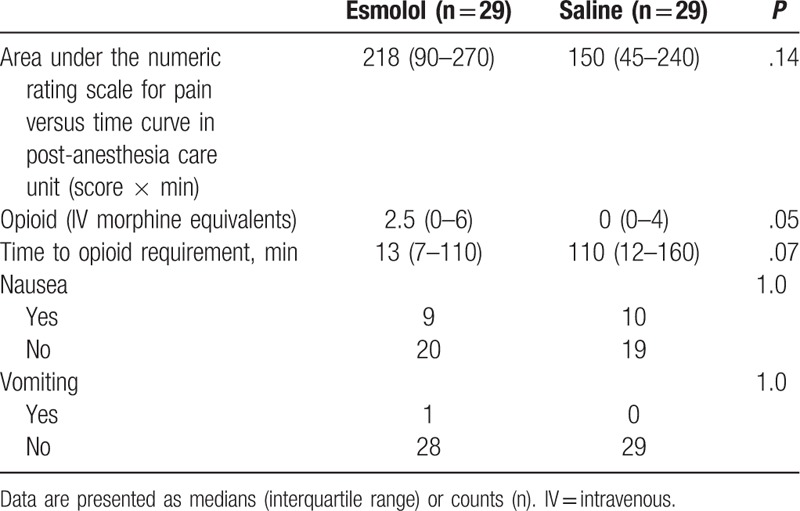
Post-anesthesia care unit data.

**Table 3:**
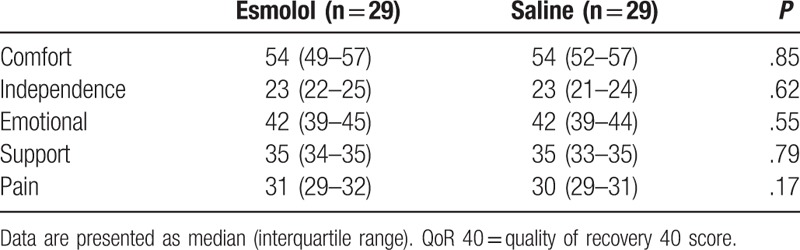
Subcomponents items of the QoR-40 according to the study groups.

None of the patients develop adverse reactions that were attributed to the study drug.

## 4 Discussion

The main finding of the current investigation was the lack of benefit of intraoperative esmolol on patient-reported quality of recovery when compared to saline after ambulatory gynecological surgery. In addition, patient receiving intraoperative esmolol did not have any improvements on outcomes in the post-anesthesia discharge unit (e.g., pain, opioid consumption, discharge readiness). Taken together, our results do not support the use of intraoperative esmolol as a clinically relevant strategy to improve postsurgical quality of recovery after ambulatory surgery.

Our results are clinically important as intraoperative esmolol has been demonstrated to reduce postsurgical pain and opioid consumption.^[26,27]^ Clinical practitioners may rely on intraoperative esmolol to enhance quality of recovery for patients undergoing outpatient surgery. The use of intraoperative esmolol may reduce the use of other strategies that have been effective in improving postsurgical outcomes after ambulatory surgery.^[28–31]^

Another important finding of our current investigation was the lack of analgesic benefits of esmolol for patients undergoing outpatient hysteroscopy. Our results confirm the concept that pharmacologic interventions to enhance recovery should be developed for specific surgical procedures.^[32–34]^ To the best of our knowledge, this is the first study to evaluate the effects of intraoperative esmolol for patients undergoing outpatient gynecologic hysteroscopies.

It is important to note that we did not give intraoperative dexamethasone as part of our study protocol. As intraoperative dexamethasone has been effective to improve several aspects of postoperative recovery (including patient-reported outcomes), we were concerned that the effects of dexamethasone would reduce any potential advantage of intraoperative systemic esmolol.^[35–38]^ It is therefore unlikely that our results would be altered if intraoperative dexamethasone had been added as a standard intervention in our study protocol.

There are several potential explanations why we did not detect an analgesic effect of esmolol on the present study. First, esmolol may not help patients undergoing hysteroscopy. This confirms the current concept that analgesic interventions should be surgery-specific.^[39,40]^ Second, it is possible that the short duration of the procedures did not allow for the full analgesic effect of esmolol. Lastly, we utilized other concurrent multimodal analgesic strategies (e.g., ketorolac and dexamethasone) and they may have reduced the potential benefits of esmolol.^[18,41]^ Future studies to confirm or refute these potential explanations are warranted.

Our study should only be considered in the context of its limitations. First, to minimize variations in recovery profiles, we examined only 1 surgical procedure and we cannot generalize our results to other types of surgeries. Although we selected a dose of esmolol similar to the one used in previous studies, we did not examine a potential dose response effect of esmolol. It is possible that greater doses of intraoperative esmolol may have a significant effect on patient-reported quality of recovery after outpatient surgery. Lastly, we did not evaluate a potential mechanistic effect of esmolol on pain thresholds, rather than we relied on previous studies in the literature.

In conclusion, we did not detect a beneficial effect of intraoperative esmolol on patient-reported quality of recovery after ambulatory hysteroscopic surgery. Our results suggest that clinical practitioner should use alternative strategies with proven efficacy to enhance recovery of patients undergoing ambulatory gynecologic hysteroscopies. In addition, our results suggest that previously thought-effective perioperative interventions may not demonstrate efficacy when patient-reported outcomes are used.

## Author contributions

**Conceptualization:** Gildasio De Oliveira, Robert Mccarthy.

**Data curation:** Gildasio De Oliveira, Robert Mccarthy.

**Formal analysis:** Gildasio De Oliveira, Mark Kendall, Robert Mccarthy.

**Funding acquisition:** Gildasio De Oliveira, Robert Mccarthy.

**Investigation:** Gildasio De Oliveira.

**Methodology:** Gildasio De Oliveira, Robert Mccarthy.

**Project administration:** Gildasio De Oliveira, Mark Kendall.

**Resources:** Gildasio De Oliveira.

**Software:** Gildasio De Oliveira.

**Supervision:** Gildasio De Oliveira.

**Validation:** Gildasio De Oliveira.

**Visualization:** Gildasio De Oliveira.

**Writing – original draft:** Gildasio De Oliveira, Mark Kendall, Robert Mccarthy.

**Writing – review & editing:** Gildasio De Oliveira, Mark Kendall, Robert Mccarthy.
